# Modulation of lactose synthesis and orexinergic‐glucose pathway by sex steroid hormones

**DOI:** 10.14814/phy2.70661

**Published:** 2025-11-16

**Authors:** Jean Claude Hakizimana, Abdullateef Isiaka Alagbonsi

**Affiliations:** ^1^ Department of Physiology, School of Medicine and Pharmacy, College of Medicine and Health Sciences University of Rwanda Huye Rwanda

**Keywords:** estrogen, glucose, lactose, orexin, progesterone, testosterone

## Abstract

Sex steroid hormones play a regulatory role in various metabolic processes, including glucose homeostasis via the orexinergic system and lactose synthesis. This review consolidates experimental findings on the mechanisms by which these hormones regulate these two pathways. A systematic search of PubMed, Scopus, and Web of Science identified 15 controlled studies involving animals and humans that investigated the effects of sex steroid hormones on both pathways. Estradiol enhanced orexin neuron excitability and increased orexin‐1 receptor expression in a cyclical, phase‐dependent manner within the orexinergic–glucose axis, promoting glucose utilization during estrogen‐dominant phases. Progesterone reduced this activity, which is consistent with the conservation of energy during the luteal phase. Testosterone diminished orexin‐A neuronal activation during glucose deficit, suggesting a suppressive effect on orexin‐driven glucose mobilization. Also, estradiol promoted lactogenesis after progesterone withdrawal, whereas progesterone sustained prepartum inhibition of α‐lactalbumin and casein gene transcription. In conclusion, the influence of sex steroid hormones on orexinergic–glucose regulation is hormone‐specific and phase‐dependent, with estradiol acting as a stimulant, progesterone as an inhibitor, and testosterone having a largely suppressive effect. The postpartum decrease in progesterone level triggers estradiol to support milk production. Studies are needed to investigate the role of sex steroid hormones on lactase expression, activity, and lactose tolerance.

## 1 INTRODUCTION

Beyond their traditional reproductive roles, sex steroid hormones such as testosterone, progesterone, and estradiol are also fundamental regulators of systemic metabolism (Kampire et al., 2025). Recent studies demonstrate their role in mammary lactose production and orexin‐mediated glucose sensing, highlighting the essentiality of sex steroid hormones for lactation and energy homeostasis. The lateral hypothalamus (LH)'s orexin (hypocretin) neurons, which are essential for metabolic regulation, combine other functions, including arousal, feeding behavior, nutritional signals, and endocrine responses (Adeghate et al., 2020; Pignatelli et al., 2022). According to recent research, circulating estradiol and metabolic risk markers are correlated with plasma orexin‐A levels, suggesting that sex steroid hormones alter the physiological functions of orexin (Messina et al., 2013).

Preclinical evidence supports sex‐specific modulation of orexin‐glucose pathways. For instance, high amounts of estradiol improve orexin neuron activity during fasting or hypoglycemia, according to studies conducted throughout the mouse estrous cycle (Funabashi et al., 2009; Kim et al., 2023). These findings link hormonal state to behavioral energy compensation and are in line with functional evidence that demonstrates estradiol‐driven restoration of orexinergic excitability following ovariectomy. Progesterone and testosterone, on the other hand, appear to influence orexinergic transmission differently, altering metabolic sensitivity and often attenuating activity. Due to its genomic and nongenomic effects in peripheral tissues, progesterone's impact on insulin resistance and glucose tolerance has gained increasing recognition (Xega & Liu, 2024). However, based on clinical data, daily glucose‐sex steroid hormone cycles indicate that changes in sex steroid hormones throughout the menstrual cycle impact hunger and glucose regulation, further linking endocrine state to orexin‐glucose control (Schieren et al., 2024).

Lactose metabolism provides another dimension of sex steroid hormonal regulation. In the mammary gland, progesterone has a temporal inhibitory effect during pregnancy, delaying the premature activation of lactogenesis until the peripartum period, while estradiol stimulates the expression of α‐lactalbumin, galactosyltransferase, and prolactin receptor (PRLR) genes (Carón & Deis, 1997; Deis et al., 1989; Lopez et al., 2016). Nonetheless, these mechanisms pertain specifically to the production of milk and do not target intestinal lactase activity or the genetic control of lactase persistence directly. The latter is primarily influenced by single‐nucleotide polymorphisms located upstream of the lactase gene (LCT) (Ingram et al., 2009). To date, no experimental studies have directly investigated whether sex steroid hormones modulate intestinal lactase expression, lactose digestion, or intolerance symptoms. Considering that lactose intolerance affects over two‐thirds of the worldwide population, with considerable variation across genetic and ethnic backgrounds (Storhaug et al., 2017), this knowledge gap is especially notable. By addressing this gap, we could find out if hormonal states such as puberty, pregnancy, menopause, or gender‐affirming hormone therapy affect how the gastrointestinal system processes dietary lactose.

The fate of most of the glucose uptake by the mammary gland is for lactose synthesis (Bautista et al., 2016). Furthermore, the rate of mammary gland's fatty acid synthesis from substrates like acetate and lactate is negligible in the absence of glucose but stimulated in the presence of glucose, while lipogenesis from glucose in combination with either of these substrates is greater than from glucose alone (Katz et al., 1974). Interestingly, starvation leads to a 50‐fold reduction of fatty acid synthesis by the mammary gland, and the optimal lipogenesis is restored 2 hours after refeeding (Anhe & Bordin, 2022). As orexin promotes food‐seeking behavior, it is plausible that modulation of glucose homeostasis through the orexinergic‐glucose pathway could influence lipogenesis, a part of lactogenesis, in the mammary gland.

Despite their shared regulation by sex steroids, the orexin‐glucose pathway and lactose metabolism are rarely studied together. This review, therefore, presents how testosterone, progesterone, and estradiol alter these pathways and disclose sex‐specific metabolic strategies using experimental data from previous studies. The review centered on two main questions: first, how do sex steroid hormones affect lactase activity, gene expression, and lactose digestion? Secondly, how do they impact the regulation of glucose metabolism and energy balance through the orexinergic pathway? By synthesizing across these pathways, the review highlights sex‐specific metabolic strategies and proposes that understanding these mechanisms could inform gender‐sensitive approaches to conditions such as gestational diabetes, obesity, and endocrine lactation failures.

## 2 METHODOLOGY

Aiming to synthesize primary research evidence on how changes in sex steroid hormones, specifically estrogen, progesterone, and testosterone, influence lactose synthesis and the orexinergic‐glucose pathway in mammalian systems, this systematic review was conducted following the PRISMA (Preferred Reporting Items for Systematic Reviews and Meta‐Analyses) 2020 guidelines (Page et al., 2021) (Appendix S1).

### 2.1 Study identification

A comprehensive literature search was performed using three electronic databases, PubMed, Scopus, and Web of Science, to identify relevant studies. Keywords and MeSH terms such as “lactose metabolism,” “lactase activity,” “galactose metabolism,” “sex steroid hormones,” “estrogen,” “testosterone,” “progesterone,” “orexin,” “hypocretin,” “glucose homeostasis,” and “energy regulation” were combined in the search strategy. Boolean operators were used to refine the results (Appendix S2). The searches were conducted during the first week of August 2025.

Studies meeting the following criteria were included in this review: they must be original, peer‐reviewed research articles published between January 1969 and July 2025. Eligible studies needed to investigate how sex steroid hormones, specifically estrogen, progesterone, or testosterone, influence the orexinergic‐glucose pathway or lactose metabolism. Outcomes of interest related to lactose metabolism included lactose synthesis, lactase activity, *LCT* expression, lactose tolerance, and galactose absorption. On the outcomes related to the orexinergic‐glucose pathway, studies examining orexin levels, receptor expression, activities, or signaling were included, provided they are associated with glucose homeostasis. Only studies conducted in mammalian systems, involving either human subjects or animal models (such as mice, rats, and primates), were considered. Furthermore, to ensure accurate data extraction and interpretation, only publications in English were included.

Review articles, meta‐analyses, editorials, commentaries, and conference abstracts lacking full‐text accessibility were not included in the study. Studies conducted in vitro were not included unless they included hormone therapies and connected the outcomes to the physiological mechanisms in vivo. Studies that used nonmammalian subjects, such as insects or zebrafish, were also disqualified because of the notable variations in their hormonal physiologies (Toso et al., 2024). Furthermore, studies that examined hormonal effects unrelated to sex steroids (such as thyroid or cortisol) or that did not evaluate outcomes directly connected to either lactose metabolism or the orexinergic‐glucose axis were excluded. Additionally, unless their design permitted a clear interpretation of sex steroid hormone‐specific effects, studies that combined hormonal interventions with confounding pharmaceutical treatments without suitable controls were not included. After screening the abstracts and titles, two impartial reviewers evaluated the entire texts of any possibly pertinent papers. During the study selection process, disagreements were settled by consensus or arbitration of a third reviewer (applicable to two articles). Risk of bias assessment was done for the studies using the SYstematic Review Center for Laboratory animal Experimentation (SYRCLE)'s RoB tool (Hooijmans et al., 2014) (Appendix S3).

### 2.2 Data extraction and synthesis

Each included study's author name, publication year, country, study design, biological model, hormone type and dosage, length of treatment, targeted pathway (lactose synthesis or orexin‐glucose), key metabolic outcomes, and principal findings were gathered using a structured data extraction form. When available, sex‐specific responses were given special consideration in the data synthesis, which was arranged by hormone type and the physiological route impacted (Appendix S4).

## 3 RESULTS

### 3.1 Outcome of the studies' screening and selection process

**FIGURE 1:**
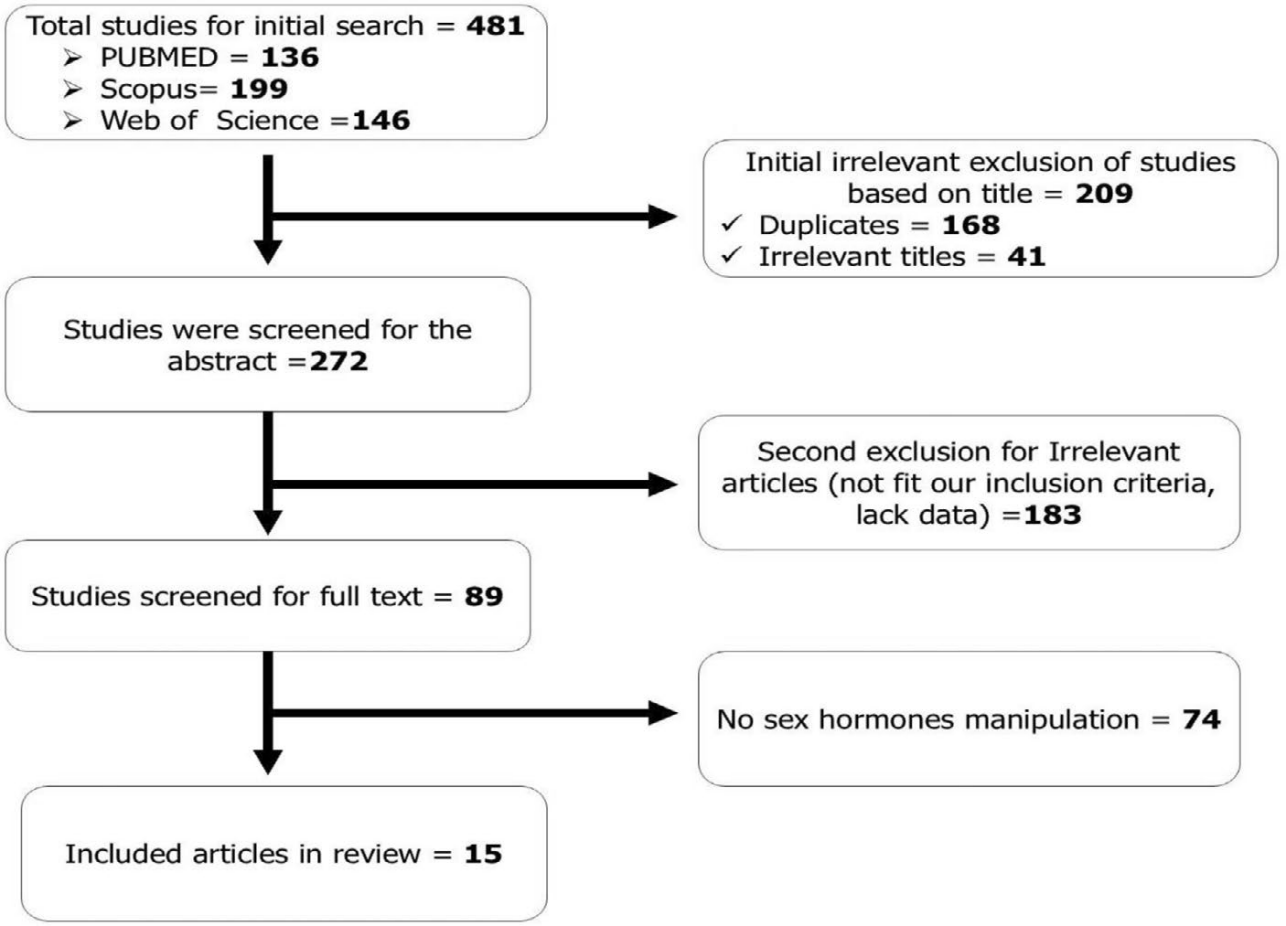
Article selection process based on PRISMA.

After a thorough screening procedure, a total of 15 primary experimental investigations were included in this review (Figure 1). Of these, five research studies examined how sex steroid hormones modulate glucose homeostasis through the orexinergic system, while ten studies assessed how sex steroid hormonal changes regulate mammary lactose production. All included studies met strict eligibility requirements: they used direct sex steroid hormone manipulation (e.g., testosterone, progesterone, and estradiol) and examined their molecular or functional effects on lactose synthesis or orexin‐mediated glucose control. One study each used tissue‐based systems from humans and cows, while the majority (13 studies) used rodent models (see details in Appendix S4). The results of the Bias of Risk assessment are also presented in the Appendix S3.

### 3.2 The role of sex steroid hormones in metabolic sensory integration

#### 3.2.1 Estradiol (E2)

Estradiol increased the activity of orexinergic neurons in the LH, particularly in response to glucose restriction, according to data from ovariectomized (OVX) models treated with E2 and female rats during stages of the menstrual cycle (Funabashi et al., 2009; Kim et al., 2023). The presence of an estradiol‐orexin‐glucose regulation axis, which promotes adaptive behavior in hypoglycemia situations, is supported by this result. Estradiol has also been demonstrated to increase orexin 1 receptor (OX1R) expression in the specific brain regions and restore normal firing rates in glucose‐inhibited neurons (Silveyra et al., 2009), both of which support energy homeostasis (Table 1).

**TABLE 1:** Modulation of the orexinergic‐glucose pathway by sex steroid hormones.

Hormone(s) tested	Study (citation)	Model/species	Animal strain and sex	Age/weight of animals	Number of subjects per group	Time of the day measurements were taken	Hormone dose, concentration, and duration	Methods used for outcome assessment	Key outcomes measured	Main finding	PMID/DOI
Estradiol (cyclic variation)	Kim et al. (2023)	Female rats (cycling)	Sprague–Dawley, female	Adult, 250–300 g	*n* = 5–8 per group	Behavioral tests: 08:00–12:00 h; Euthanasia: 08:00–10:00 h	Natural cyclic variation, no exogenous dose	Electrophysiology, IHC, behavioral assays	Orexin neuron activity; glucose‐dependent behavior	Estradiol modulates orexinergic neurons across the estrous cycle, affects glucose‐dependent behavior, and arousal/feeding responses	PMID: 36571628 DOI: 10.1007/s00213‐022‐06296‐1
Estradiol (OVX and E2 replacement)	Funabashi et al. (2009)	Ovariectomized rats	Wistar, female	Adult, 250–300 g	*n* = 3–5 per group	Started at 11:00 h	Estradiol benzoate 2 μg in 0.1 mL oil s.c., 2 days after OVX	pCREB activation/IHC	Orexin neuron response to fasting/hypoglycemia	Estradiol restoration of orexin neuron response to fasting/hypoglycemia	PMID: 19616070 DOI: 10.1016/j.neulet.2009.07.035
Estradiol and progesterone	Silveyra et al. (2009)	Rats (OVX + treatments)	Sprague–Dawley, male and female	Adult, 200–250 g	*n* = 4–6 per group	10:00 h or 20:00 h	Estradiol 0.1 mg/kg s.c., progesterone 1 mg/rat s.c., single or sequential	ISH and qPCR, and IHC for OX1R	OX1R expression (hypothalamic nuclei)	Estradiol increases OX1R expression; progesterone given after estradiol attenuates this upregulation	PMID: 19699765 DOI: 10.1016/j.regpep.2009.08.002
Testosterone (endogenous)	Takamata et al. (2022)	Male rats	Wistar, male	10–11 weeks, 270–300 g	*n* = 6–8 per group	Light phase (10:00–16:00)	Endogenous, no exogenous	Neuronal activation markers/IHC	Orexin‐A activation during glucose deficit	Testosterone reduces the activation of orexin‐A neurons in response to glucose deficit	PMCID: PMC8950295 DOI: 10.3390/nu14061235
Ovarian steroids (E2 + P4)	Pu et al. (1998)	Female rats	Sprague–Dawley, female	Adult, 180–200 g	*n* = 4–5 per group	Not specified	Estradiol benzoate 1 μg/rat s.c., progesterone 1 mg/rat s.c., for 3 days	Neuroendocrine assays (luteinizing hormone measurement)	Pituitary luteinizing hormone release in response to orexin stimulation	Ovarian hormone status modulates luteinizing hormone response to orexin	PMID: 9879756 DOI: 10.1016/s0167‐0115(98)00128‐1

Abbreviations: E2, estradiol; IHC, immunohistochemistry; ISH, in‐situ hybridization; mRNA, messenger ribonucleic acid; OVX, ovariectomy; OX1R, orexin‐1 receptor; P4, progesterone; pCREB, phosphorylated cyclic adenosine monophosphate response element‐binding protein; PRLR, prolactin receptor; qPCR, quantitative polymerase chain reaction; s.c., subcutaneous.

Available studies also support the lactogenic effect of estradiol, acting both centrally and peripherally. By promoting the development of PRLRs and lactose‐synthesizing enzymes like α‐lactalbumin, estradiol prepares breast tissue for lactogenesis. Estradiol works in concert with prolactin to promote the start of milk production in the later stages of pregnancy and the postpartum period (Carón & Deis, 1997; McGuire, 1969). Estradiol, therefore, functions as a pro‐metabolic hormone in both the central and peripheral systems, improving the brain's ability to sense nutrients and the mammary gland's ability to provide them (Table 2).

**TABLE 2:** Modulation of lactose homeostasis by sex steroid hormones.

Hormone(s) tested	Study (citation)	Model/species	Animal strain and sex	Age/weight of animals	Number of subjects per group	Time of day measurements were taken	Hormone dose, concentration, and duration	Methods used for outcome assessment	Key outcomes measured	Main finding	PMID/DOI
Estradiol implants; progesterone role	Carón and Deis (1997)	Virgin rats (arcuate nucleus implants)	Wistar, female	3–4 months, 200–220 g	Not specified, typical 5–6	Not specified	17β‐estradiol implants in the arcuate nucleus	Mammary gland assays; milk protein markers	Lactogenesis onset (mammary markers)	Estradiol implants in the arcuate nucleus induce lactogenesis; progesterone modulates/times lactogenesis	PMID: 9488101 DOI: 10.1016/s0024‐3205(97)01091‐6
Progesterone antagonist (mifepristone) + prolactin (suckling)	Deis et al. (1989)	Pregnant rats	Wistar, female	Virgin, 180–200 g	*n* = 8 per group	Not specified	Mifepristone 2 mg/kg s.c.	Hormone assays; milk markers	Prolactin release; timing of lactogenesis	Suckling‐induced prolactin release potentiates mifepristone‐induced lactogenesis earlier lactose/milk production	PMID: 2621690 DOI: 10.1530/jrf.0.0870147
Progesterone (endogenous/experimental)	López‐Fontana et al. (2012)	Pregnant rodents	Wistar rats and C57BL/6 mice, female	Adult virgin mated	*n* = 5–6 per group	Decapitated at 1000 h	Endogenous/experimental progesterone	Milk assays; some gene expression measures	Milk yield; lactogenesis inhibition markers	Progesterone inhibits lactogenesis in mid‐to‐late gestation; withdrawal is required for lactogenesis II	PMID: 22697120 DOI: 10.1071/RD11160
Estradiol and placental lactogen	Bussmann et al. (1983)	Rats	Wistar, female	Virgin, 180–200 g	*n* = 6–8 per group	Decapitated 28 h after surgery	Estradiol benzoate 1 μg/rat, placental lactogen 0.5 mg/rat	Mammary gene expression assays	Milk protein & lactose‐related gene expression	Synergistic effect of E2 and placental lactogen on milk protein and lactose‐related genes	PMID: 6414540 DOI: 10.1095/biolreprod29.3.535
17β‐Estradiol (exogenous)	Delbecchi et al. (2005)	Dairy cows (mid‐lactation)	Holstein, female	Mid‐lactation, DIM 173 ± 12	n = 8	Not specified	0.021 mg/kg B.W. per day for 7 days	Milk production records; gene expression panels	Milk yield; mammary gene expression (general)	Decreased milk yield; altered expression of milk protein/involution‐related genes; lactose‐specific genes were not assessed	https://doi.org/10.1016/j.livprodsci.2005.10.010
Estradiol (and lactation hormones)	Bolander and Topper (1980)	Mouse mammary tissue/explants	C3H/HeN mice, female	Mid‐pregnant	Explants	Not specified	10 ng/mL estradiol‐17β	mRNA assays/molecular biology	Casein and α‐lactalbumin mRNA	Hormonal regulation of casein and α‐lactalbumin mRNA (supports estradiol role in lactation gene expression)	PMID: 6766381 DOI: 10.1210/endo‐106‐2‐490
Progesterone	Murphy et al. (1973)	Pregnant rats	Hooded Norway, female	Primiparous	Not specified	Not specified	Progesterone	Biochemical assays	Lactose biosynthesis measures	Progesterone suppresses lactose biosynthesis during pregnancy	PMID: 4362333 DOI: 10.1042/bj1361105
Estradiol (hormonal regulation)	Palmiter (1969)	Mouse mammary gland	C3H/HeN, female	Mid‐pregnant	Explants	Not specified	Not specified	Enzyme activity assays	Lactose synthetase regulation/activity	Hormonal regulation of lactose synthetase in the mammary gland	PMID: 5808320 DOI: 10.1042/bj1130409
Estradiol	McGuire (1969)	Human breast explants (in vitro)	Human, female	Not specified	Explants	Not specified	Estradiol	In vitro tissue assays	α‐lactalbumin synthesis	Estrogen induction of α‐lactalbumin synthesis in human breast tissue	PMID: 4896309 DOI: 10.1126/science.165.3897.1013
Estradiol	Harigaya et al. (1978)	Pregnant mice (OVX contexts)	KA strain, female	Pregnant	Not specified	Not specified	Estradiol after OVX	Receptor assays (molecular assays)	Prolactin receptor (PRLR) expression in the mammary gland	Estradiol induces PRLR expression, enabling prolactin responsiveness for lactogenesis	PMID: 208831 DOI: 10.1507/endocrj1954.25.157

Abbreviations: B.W., body weight; DIM, days in milk; E2, estradiol; mRNA, messenger ribonucleic acid; OVX, ovariectomy; P4, progesterone; PRLR, prolactin receptor.

#### 3.2.2 Progesterone and combined hormonal state (E2 and P4)

Progesterone demonstrated context‐dependent modulation of orexin‐related activity, primarily when tested alongside estradiol. Variations in ovarian steroids (E2 and P4) during mid‐to‐late trimester rat models affected the expression of orexin receptors and the release of orexin by the LH in a region‐specific manner (Pu et al., 1998; Silveyra et al., 2009). Ovarian steroids (E2 and P4) influenced hypothalamic–pituitary neuroendocrine responsiveness, especially luteinizing hormone release, in a way that depends on the hormonal environment. Estradiol priming enhanced hypothalamic sensitivity to orexin input, while the addition of progesterone altered the response's amplitude and timing (Pu et al., 1998). This suggests that the metabolic regulation mediated by orexin, including glucose‐related activity in the hypothalamus, varies with the phase; it is heightened during estradiol‐dominant periods and modulated (sometimes reduced) during progesterone‐dominant phases.

Furthermore, estradiol increases OX1R expression in key areas of the hypothalamus, and progesterone reduces this receptor's expression to baseline levels after estradiol priming (Silveyra et al., 2009). This interaction creates a cyclical pattern: in the follicular or ovulatory phase, increased estradiol may boost orexinergic tone, which may enhance glucose sensing and uptake; during the luteal phase, elevated progesterone levels may dampen this activation and shift the metabolic set‐point toward energy conservation. These findings imply that progesterone modifies estradiol‐driven orexinergic activity and contributes to sex steroid hormone rhythmicity in energy regulation, even though it is not an independent signal to orexin neurons.

Progesterone functions as a physiological inhibitor of lactose synthesis in the mammary gland, inhibiting the expression of α‐lactalbumin and postponing the start of complete lactogenesis until parturition (López‐Fontana et al., 2012). The key to starting milk production is not its presence but rather its departure. Thus, although progesterone plays a part in the hormonal priming of lactogenesis, its main function is inhibitory, which is in line with its ability to modulate and temporally constrain both central and peripheral metabolic pathways.

#### 3.2.3 Testosterone

A study used intact male rats to examine the function of testosterone in orexinergic‐glucose pathways. The activation of orexin‐A neurons in response to glucose restriction was consistently inhibited by testosterone in the animals (Takamata et al., 2022). The reduced behavioral and physiological responses to hypoglycemia seen in males as opposed to females may be attributed in part to this inhibition of orexin reactivity. These results highlight a key glucose‐sensing mechanism that is sex‐dimorphic and may be driven by androgen signaling.

None of the reviewed studies investigated testosterone's effect on lactogenic processes, and unlike estrogen, it has not been directly connected to the control of lactose synthesis. Testosterone's suppression of central orexinergic transmission, however, may indirectly affect metabolic preparedness and the stress response during energy scarcity or male parental care in biparental species where males do not lactate.

### 3.3 A hormonal bridge between energy balance and milk production

When combined, the data show a logical hormonal pattern where estradiol functions as a major metabolic facilitator by improving peripheral lactose synthesis and central glucose sensing. Progesterone, on the other hand, works in concert with estradiol to fine‐tune hypothalamic responses and inhibit lactogenic processes in particular phases, while testosterone limits central glucose adaptation through orexinergic suppression. These results lend credence to the idea that sex steroid hormones' control of energy balance is integrative, balancing physiological preparedness for nursing and reproduction with metabolic cues acquired from the brain.

## 4 DISCUSSION

The intricate and interconnected functions of sex steroid hormones (testosterone, progesterone, and estrogen) in controlling lactogenesis and orexinergic‐glucose transmission are highlighted in this systematic review. Significant patterns of modulation throughout the central and peripheral metabolic axes are revealed by the evidence compiled from 15 main investigations, highlighting hormone‐dependent and sex‐specific physiological mechanisms that are relevant to both experimentation and clinical settings.

### 4.1 Major findings and mechanistic interpretation

By activating orexinergic neurons in the LH, estradiol became the primary enhancer of central glucose sensing. In ovariectomized rats treated with estradiol, these neurons, which are known to mediate arousal, energy intake, and glucose detection, reacted more strongly to hypoglycemia than did controls (Funabashi et al., 2009; Kim et al., 2023). Estradiol may improve adaptive behavior in response to caloric deprivation by increasing orexin‐A production and firing rate through pathways that are dependent on the estrogen receptor α (ERα). This is consistent with research on female humans, which shows that enhanced insulin sensitivity and glucose elimination are correlated with higher follicular phase estrogen levels (Mauvais‐Jarvis et al., 2013).

Beyond central glucose sensing, estradiol also primes lactose synthesis by promoting the expression of *α‐lactalbumin* and PRLR genes in the mammary epithelial cells (Bolander & Topper, 1980; Delbecchi et al., 2005; Harigaya et al., 1978; McGuire, 1969; Palmiter, 1969). These findings are physiologically coherent, since complete lactogenesis is triggered by the withdrawal of progesterone and the rise in prolactin, while estradiol primes mammary tissue in late gestation (Carón & Deis, 1997; Deis et al., 1989). Importantly, this means estradiol acts in dual roles: maintaining maternal energy sensing and supporting lactogenic readiness, thereby highlighting the coordinated role of estradiol in energy and lactose metabolic pathways. As the fate of the majority of glucose taken up by the mammary gland is for lactose production, estrogen likely promotes glucose uptake and utilization by the mammary gland to drive lactogenesis. Notably, no study investigated the effect of estrogens on either lactose digestion and lactase expression or lactase persistence and lactose intolerance.

Conversely, during glucose restriction, testosterone demonstrated an inhibitory effect on orexinergic neurons. Under fasting conditions, male rats with higher levels of testosterone in their blood showed less activation of orexin‐A neurons in the LH (Takamata et al., 2022). This suggests that testosterone may reduce the central neuroendocrine response to metabolic stress, but the mechanism remains unclear. Given that only one study directly addressed testosterone's effects on the orexinergic–glucose pathway, this result should be interpreted cautiously. It is also worth noting that females produce testosterone, albeit at lower levels than males, but none of the outlined studies assessed testosterone levels or its potential effects on these pathways in female models.

**FIGURE 2:**
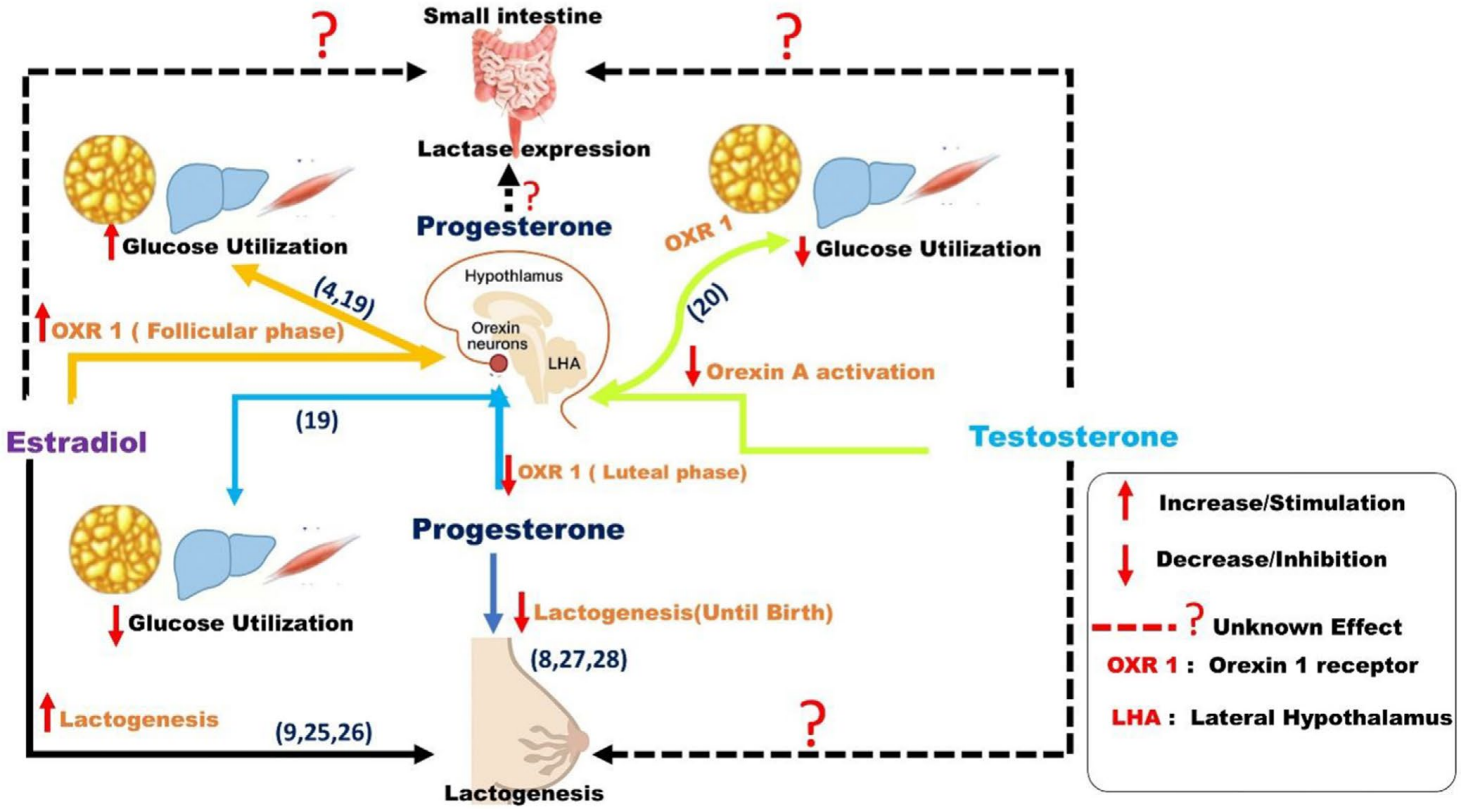
Proposed model of orexin A and orexin 1 receptor (OX1R) signaling in the lateral hypothalamic area (LHA), regulating glucose utilization and lactogenesis in response to estradiol and progesterone across different female reproductive phases (follicular and luteal phases), and testosterone in males. Estradiol enhances orexinergic activity in the lateral hypothalamic area (LHA), promoting glucose utilization and preparing mammary tissue for lactose synthesis (possibly via enhanced glucose utilization) during the follicular phase. Conversely, progesterone inhibits orexin signaling in the luteal phase, delaying lactogenesis (possibly via reduced glucose utilization) until parturition. Testosterone decreases orexin‐A activation during hypoglycemia, impacting glucose responses, while its effects on lactogenesis are uncertain. Overall, these hormones play a crucial role in balancing energy with reproductive and lactational functions.

Progesterone, when coadministered with estradiol, showed context‐dependent modulation of both orexin signaling and lactogenic activity. In hypothalamic circuits, progesterone moderated the increase in OX1R expression caused by estradiol, which lowered orexinergic excitability during phases dominated by progesterone (Silveyra et al., 2009). In mammary models, progesterone inhibits the transcription of α‐lactalbumin and casein genes during mid to late gestation, delaying lactose production until parturition (Carón & Deis, 1997; López‐Fontana et al., 2012). This confirms progesterone's established roles as a physiological inhibitor of lactogenesis before birth, although mechanistic conclusions remain tentative given the small number of available studies (Figure 2).

The notion that sex steroid hormones mediate energy metabolism through peripheral and hypothalamic signaling networks is supported by a number of studies in addition to the major studies that are included. For instance, progesterone inhibits insulin receptor substrate (IRS‐1) signaling, which may lead to insulin resistance during pregnancy (Yan et al., 2019), while estrogen increases GLUT4 translocation in adipocytes and the liver (Campello et al., 2017). Additionally, it has been suggested that sex steroids have a broad integrative function in homeostasis since orexin neurons have been linked to sleep, thermoregulation, and reward systems that are all impacted by hormonal cycles.

While estradiol maintains proper maternal energy sensing and simultaneously primes the mammary gland for lactose synthesis, this dual function has rarely been conceptualized together in the literature. To our knowledge, this review is the first to systematically align sex steroid (estradiol, progesterone, and testosterone) effects on both lactogenic processes and orexin‐mediated glucose regulation under the same hormonal framework.

### 4.2 Clinical implications

The review's conclusions have several important implications for treating metabolic diseases and reproductive health. Fluctuations in estradiol during the menstrual cycle, pregnancy, and menopause may influence glycemic control and the activity of orexinergic neurons and other hypothalamic nutrient‐sensing circuits in women. Variations in awareness of hypoglycemia, food behaviors, and energy expenditure across hormonal states could be partially explained by the findings in this review. Since estradiol impacts glucose homeostasis through both central and peripheral mechanisms, it may be a target for metabolic treatments, especially in premenopausal or perimenopausal women.

Additionally, changes in orexin signaling and glucose sensitivity might occur in endocrine disorders such as hypogonadism or polycystic ovarian syndrome (PCOS), which are often marked by altered sex steroid levels. This creates new considerations for customized metabolic therapy for these populations. Moreover, the findings have broader implications for transgender medicine. Patients undergoing gender‐affirming hormone therapy may experience shifts in energy balance, insulin sensitivity, or gastrointestinal tolerance to lactose‐containing meals.

Despite these potential effects, no clinical studies have thoroughly examined how sex steroid hormone treatments influence lactose digestion. It is worth noting that there is currently no information on the modulation of *LCT* gene expression or lactase activity by sex steroid hormones. Available studies have focused only on lactogenesis in the mammary gland, ignoring the expression of the *LCT* gene, the activity of the lactase enzyme, and the digestion of lactose in the gut. Thus, a sex difference in the well‐established lactose intolerance or lactase persistence is yet to be appreciated. This underscores the need for more inclusive, hormone‐aware metabolic research that serves diverse populations and addresses a significant gap in current data.

### 4.3 Study strengths

This review has several noteworthy advantages that contribute to the existing body of knowledge. It is the first to combine peripheral lactogenesis and central orexin‐mediated glucose sensing, two facets of metabolic regulation, under the umbrella of sex steroid hormone modulation. To enhance the robustness of its findings, the review implemented a strict inclusion protocol, focusing on original studies with validated hormonal interventions to improve internal validity and reduce bias. Finally, the dual‐pathway approach provides a distinct conceptual framework that emphasizes how sex steroid hormones coordinate the neuroendocrine and lactational pathways to match energy supply with reproductive function. The review suggests that female sex hormones modulate orexinergic‐glucose regulation to adjust lactogenesis based on the reproductive phase of the mother. This integrative lens clarifies estradiol's cross‐system effects, draws attention to the underappreciated influence of progesterone and testosterone, and identifies clear evidence gaps, such as the role of sex steroid hormones in post‐weaning lactose digestion. By doing so, this review lays a foundation for multidisciplinary, translationally relevant research.

### 4.4 Limitations and future direction

Several limitations were acknowledged in this study. First, much of the evidence (14 out of 15) derives from animal studies, particularly rodents (13 studies). Although experimentally useful, they may not capture the complexity of human hormonal cycles and the physiology of energy balance–lactogenesis pathways. Second, most studies employed exogenous hormone administration at pharmacological doses, rather than physiological fluctuations occurring during puberty, menstrual cycles, pregnancy, or menopause; so, this constrains the translational relevance of the findings. Third, there is little evidence base for some hormones; testosterone and progesterone conclusions were drawn from a single study each in the orexinergic–glucose context, limiting mechanistic certainty. Fourth, our review focused on sex steroid hormones (estradiol, progesterone, and testosterone); while nonsteroid key lactogenic hormones such as prolactin and oxytocin, and potential modulators like phytoestrogens, were not included, despite their known impacts on both lactation and glucose metabolism.

We recommend a future study that will encompass all reproductive hormones (steroids and peptides) for a wider insight. Fifth, the search period (1969–2025) was intentionally broad to capture both foundational and contemporary literature, but this also increased the screening workload and risked terminology drift. Lastly, the inclusion of PubMed, Scopus, and Web of Science minimized bias, though reliance on three databases may have excluded some gray literature.

Looking forward, research priorities should include longitudinal, sex‐stratified human studies to better capture endogenous hormonal fluctuations, integrative studies linking lactogenesis, orexinergic‐glucose activity, dietary factors, and cross‐population analyses that incorporate genetic diversity in lactase persistence. Systemic biology approaches modeling brain‐gut endocrine interactions may offer the most comprehensive way to understand these dual pathways. Ultimately, clarifying these hormonal mechanisms could inform gender‐sensitive dietary guidelines and interventions for metabolic diseases, gestational diabetes, and breastfeeding failure.

## 5 CONCLUSION

Sex steroid hormones are vital in controlling nutritional readiness and energy balance. Estradiol boosts orexinergic‐glucose sensing in the hypothalamus and prepares mammary tissue for lactogenesis. Progesterone influences when lactation begins, delaying lactose production until birth, and reduces estradiol‐driven orexin signaling. Testosterone may inhibit the brain's response to metabolic stress, showing a sex‐based difference in orexin control. However, evidence is limited, primarily from animal studies and small human research, making it challenging to fully understand human metabolic changes in glucose homeostasis and lactogenesis, particularly concerning hormonal changes during puberty, pregnancy, menopause, and hormone therapy. Future research should focus on human studies, including key lactation regulators such as prolactin and oxytocin, and consider genetic diversity in lactase persistence.

## AUTHOR CONTRIBUTIONS

JH participated in the data curation, formal analysis, investigation, methodology, software, validation, visualization, and writing original draft. AIA participated in the conceptualization, data curation, formal analysis, investigation, methodology, project administration, software, supervision, validation, visualization, writing original draft, writing review, and editing.

## FUNDING INFORMATION

The authors declare that no financial support was received for the research, authorship, and/or publication of this article.

## CONFLICT OF INTEREST STATEMENT

The authors declare that the research was conducted in the absence of any commercial or financial relationships that could be construed as a potential conflict of interest.

## Supporting information


Appendices S1–S4.
