# Supplementary material for: Modulation of lactose synthesis and orexinergic‐glucose pathway by sex steroid hormones
Source: Physiol Rep. 2025 Nov 16;13(22):e70661. doi: 10.14814/phy2.70661 (PMC12620397; doi:10.14814/phy2.70661)
Supplement: Supplementary file 1 — Appendices S1–S4. [file PHY2-13-e70661-s001.zip › Supplementary file S1.docx]

| **Section and Topic** | **Item #** | **Checklist item** | Location where item is reported |
| --- | --- | --- | --- |
| **TITLE** | | |  |
| Title | 1 | Identify the report as a systematic review. | *Modulation of lactose synthesis and orexinergic-glucose pathway by sex steroid hormones: A Systematic Review* |
| **ABSTRACT** | | |  |
| Abstract | 2 | See the PRISMA 2020 for Abstracts checklist. | **Introduction:** Sex steroid hormones like estradiol, progesterone, and testosterone play a regulatory role in various metabolic processes, including glucose homeostasis via the orexinergic system and lactose synthesis. This review consolidates experimental findings on the mechanisms by which these hormones regulate these two important metabolic pathways.  **Methods:** Up to July 2025, a systematic search of PubMed, Scopus, and Web of Science identified 15 controlled studies involving animals and humans that investigated the effects of sex steroid hormones on lactose synthesis and the orexinergic-glucose pathway.  **Results:** Estradiol enhanced orexin neuron excitability and increased orexin-1 receptor expression in a cyclical, phase-dependent manner within the orexinergic-glucose axis, promoting glucose utilization during estrogen-dominant phases. Progesterone reduced this activity, which is consistent with the conservation of energy during the luteal phase. Testosterone diminished orexin-A neuronal activation during glucose deficit, suggesting a suppressive effect on orexin-driven glucose mobilization. Also, estradiol promoted lactogenesis after progesterone withdrawal, whereas progesterone sustained prepartum inhibition of α-lactalbumin and casein gene transcription. However, no study directly analyzed sex steroid hormone effects on intestinal lactase expression or lactose tolerance.  **Conclusion:** The influence of sex steroid hormones on orexinergic-glucose regulation is hormone-specific and phase-dependent, with estradiol acting as a stimulant, progesterone as an inhibitor, and testosterone having a largely suppressive effect. A postpartum decrease in progesterone level triggers estradiol to support milk production. Studies are needed to investigate the role of sex steroid hormones on lactase expression, activity, and lactose tolerance. |
| **INTRODUCTION** | | |  |
| Rationale | 3 | Describe the rationale for the review in the context of existing knowledge. | Sex steroid hormones, including estradiol, progesterone, and testosterone, are central regulators of energy balance and metabolic processes. Estradiol has been shown to enhance insulin sensitivity and modulate hypothalamic orexinergic neurons involved in glucose regulation, while progesterone and testosterone can attenuate these effects. Beyond glucose metabolism, sex steroid hormones also play pivotal roles in lactogenesis and may influence lactose synthesis by regulating lactation-related genes such as α-lactalbumin. However, despite established links between sex steroid hormones and individual metabolic pathways, no prior synthesis has integrated evidence on their dual impact on glucose–orexinergic regulation and lactose synthesis. This review addresses this gap by systematically evaluating experimental studies, thereby providing a comprehensive perspective on hormonal modulation of these interconnected systems. |
| Objectives | 4 | Provide an explicit statement of the objective(s) or question(s) the review addresses. | To synthesize experimental evidence on estradiol, progesterone, and testosterone modulation of (i) orexinergic-glucose pathways, and (ii) lactose metabolism. |
| **METHODS** | | |  |
| Eligibility criteria | 5 | Specify the inclusion and exclusion criteria for the review and how studies were grouped for the syntheses. | - **Inclusion criteria:** Primary experimental studies (animal or human) that manipulated sex steroid hormones (endogenous or exogenous) and assessed outcomes related to glucose–orexinergic pathways (orexin activity, glucose uptake, gluconeogenesis) and/or lactose metabolism (lactogenesis, lactose-related gene expression, lactase activity). Studies had to report measurable physiological, biochemical, or molecular endpoints. - **Exclusion criteria:** Reviews, meta-analyses, editorials, conference abstracts without full data, purely observational studies, studies without sex steroid hormone manipulation, or studies focusing solely on unrelated metabolic pathways. |
| Information sources | 6 | Specify all databases, registers, websites, organisations, reference lists and other sources searched or consulted to identify studies. Specify the date when each source was last searched or consulted. | We searched PubMed, Scopus, and Web of Science for eligible studies. Additionally, reference lists of included articles were manually screened, and targeted searches of relevant journals were performed. All databases were last searched on July 25, 2025. |
| Search strategy | 7 | Present the full search strategies for all databases, registers and websites, including any filters and limits used. | A combination of controlled vocabulary (MeSH terms) and free-text keywords was used, covering three domains: *sex steroid hormones* (e.g., "estradiol," "progesterone," "testosterone"), *target pathways* (e.g., "orexin," "hypocretin," "glucose homeostasis," "gluconeogenesis"), and *lactose/lactase* (e.g., "lactose metabolism," "lactase persistence," "lactase gene expression," "lactogenesis"). Boolean operators (“AND,” “OR”) were applied. An example PubMed search string is provided in Supplementary File 1. No filters were applied except for language (English) and publication type (full-text primary research). |
| Selection process | 8 | Specify the methods used to decide whether a study met the inclusion criteria of the review, including how many reviewers screened each record and each report retrieved, whether they worked independently, and if applicable, details of automation tools used in the process. | Two independent reviewers screened titles and abstracts for relevance against predefined inclusion criteria. Full texts of potentially eligible studies were retrieved and assessed in duplicate. Discrepancies were resolved by discussion and consensus. No automation tools were used. |
| Data collection process | 9 | Specify the methods used to collect data from reports, including how many reviewers collected data from each report, whether they worked independently, any processes for obtaining or confirming data from study investigators, and if applicable, details of automation tools used in the process. | Two reviewers independently extracted data using a standardized extraction sheet, recording study design, hormone(s) tested, experimental model, intervention details, outcomes, and key findings. Data discrepancies were resolved through consensus. No automation tools were used. In cases of missing data, study authors were contacted via email; if no response was received within four weeks, the data were recorded as unavailable. |
| Data items | 10a | List and define all outcomes for which data were sought. Specify whether all results that were compatible with each outcome domain in each study were sought (e.g. for all measures, time points, analyses), and if not, the methods used to decide which results to collect. | **Primary outcomes included:**   - Glucose–orexinergic pathways: orexin neuron activity, orexin receptor expression, hypothalamic nutrient-sensing changes, glucose uptake, insulin sensitivity, gluconeogenesis. - Lactose metabolism: lactogenesis onset, lactose yield, α-lactalbumin expression, other lactose-related gene expression, lactase activity, lactose tolerance status.   All reported time points and measures for these outcomes were sought. If multiple measures existed for a single outcome domain, preference was given to physiologically validated assays and primary endpoints defined by study authors. |
|  | 10b | List and define all other variables for which data were sought (e.g. participant and intervention characteristics, funding sources). Describe any assumptions made about any missing or unclear information. | Additional data included species, strain, sex, sample size, intervention type (endogenous variation vs. exogenous administration), study funding, and reported conflicts of interest. When unclear, hormone doses were assumed to be in the range reported in the methods section; missing experimental details were noted. |
| Study risk of bias assessment | 11 | Specify the methods used to assess risk of bias in the included studies, including details of the tool(s) used, how many reviewers assessed each study and whether they worked independently, and if applicable, details of automation tools used in the process. | Animal studies were evaluated using SYRCLE’s Risk of Bias tool, and human studies were assessed using the Cochrane RoB 2 tool. Two reviewers conducted assessments independently; disagreements were resolved by discussion. No automation tools were used. |
| Effect measures | 12 | Specify for each outcome the effect measure(s) (e.g. risk ratio, mean difference) used in the synthesis or presentation of results. | Given the heterogeneity of models and outcome measures, no single quantitative effect measure was applied across all studies. Otherwise, the direction and magnitude of effects were described narratively. |
| Synthesis methods | 13a | Describe the processes used to decide which studies were eligible for each synthesis (e.g. tabulating the study intervention characteristics and comparing against the planned groups for each synthesis (item #5)). | Synthesis methods: Narrative synthesis grouped studies into three main categories based on the primary hormone examined: (i) Estradiol-related modulation, (ii) Progesterone-related modulation, and (iii) Testosterone-related modulation. Within each hormone group, studies were further sub-grouped by metabolic pathway focus—glucose–orexinergic or lactose metabolism. |
|  | 13b | Describe any methods required to prepare the data for presentation or synthesis, such as handling of missing summary statistics, or data conversions. | **Eligibility determination for each synthesis**  After completion of full-text screening, all studies meeting the predefined inclusion criteria were entered into an evidence-mapping table that listed:   - Study identifiers (authors, year) - Population characteristics (species, sex, age, physiological status) - Hormone(s) tested (estradiol, progesterone, testosterone, or combinations) - Intervention characteristics (hormone dose, route, and duration) - Primary outcomes (glucose–orexinergic pathway measures, lactose metabolism outcomes) - Secondary outcomes (gene expression, receptor distribution, biochemical markers)   We then compared these characteristics against our planned synthesis groupings:   1. By hormone type – Estradiol, Progesterone, Testosterone. 2. By metabolic focus – (i) Glucose–orexinergic modulation, (ii) Lactose metabolism modulation. 3. By experimental model – Human studies, rodent models, other mammals.   Process:   - Two reviewers independently examined the extraction table to determine if each study aligned with at least one planned synthesis subgroup. - Studies were included in a synthesis if both the intervention and outcome corresponded to one of the predefined groups (e.g., estradiol administration to orexin neuron activity to glucose regulation). - When studies examined multiple hormones or pathways, they were classified into multiple relevant synthesis groups. - Studies with insufficient detail on intervention characteristics or lacking quantifiable outcome measures for either pathway were excluded from synthesis but recorded in an “Excluded after eligibility check” log with the reason for exclusion.   This process ensured that each synthesis incorporated only studies that were methodologically aligned and directly relevant to the pathway and hormone category under investigation. |
|  | 13c | Describe any methods used to tabulate or visually display results of individual studies and syntheses. | **Tabulation and Visualisation Methods**  To ensure clarity and facilitate comparison across studies, we used structured tabulation and visual representation approaches for both individual study results and synthesis summaries.  1. Tabulation of individual study results   - For each included study, we extracted and presented the following in structured tables:   - Citation (authors, year, PMID/DOI)   - Hormone(s) tested   - Experimental model (species, sex, physiological status)   - Intervention details (dose, route, timing)   - Primary outcome(s) and key findings relevant to glucose–orexinergic modulation and/or lactose metabolism   - Notes on methodological quality and any reported limitations - Tables were grouped by hormone category (Estradiol, Progesterone, Testosterone) and then subdivided by metabolic focus (glucose–orexinergic vs. lactose synthesis). - This grouping allowed rapid identification of hormone–pathway relationships.   2. Visual displays for synthesis   - Flow diagram: A PRISMA 2020-compliant flowchart was used to show the number of records identified, screened, excluded, and included, as well as reasons for exclusion. - Schematic diagrams: Created to depict physiological pathways illustrating how each sex steroid hormone modulates glucose–orexinergic circuits and/or lactose synthesis, based on the included evidence. - Summary figures: Bar charts and bubble plots were used to indicate the distribution of studies by hormone type, metabolic pathway, and model organism.   3. Comparative tables for synthesis   - Separate synthesis tables were prepared to show aggregated findings across studies, including:   - Number of studies reporting increased, decreased, or no change in a given outcome   - Species and experimental model breakdown   - Consistency of results across methodological designs - Where appropriate, synthesis tables included direction of effect icons (↑ increase, ↓ decrease, ↔ no significant change) to enhance visual interpretation.   4. Software used   - Tabulations were prepared in Microsoft Excel and formatted in Word for inclusion in the manuscript. - Figures and schematic diagrams were generated using Microsoft PowerPoint. |
|  | 13d | Describe any methods used to synthesize results and provide a rationale for the choice(s). If meta-analysis was performed, describe the model(s), method(s) to identify the presence and extent of statistical heterogeneity, and software package(s) used. | **Synthesis Methods and Rationale**  Given the heterogeneity of the included studies in terms of species, experimental designs, hormonal interventions (dose, route, and duration), and outcome measures, we employed a narrative synthesis approach rather than a meta-analysis.  1. Rationale for synthesis choice   - The studies varied widely in experimental models (rodents, livestock, humans), hormone administration protocols (physiological vs. pharmacological doses), and measured outcomes (e.g., orexin neuron firing rate, glucose uptake assays, lactase gene expression). - Due to this high degree of methodological and outcome heterogeneity, statistical pooling via meta-analysis was not appropriate, as combining effect sizes would not yield meaningful or interpretable results. - A qualitative, thematic grouping was therefore chosen to preserve biological context and mechanistic insight.   2. Narrative synthesis approach   - Studies were first grouped by hormone type (estradiol, progesterone, testosterone). - Within each hormone category, studies were further divided into two mechanistic pathways:   1. Glucose–orexinergic modulation   2. Lactose synthesis modulation - For each subgroup, we compared study findings in terms of direction of effect (↑ increase, ↓ decrease, ↔ no change) and discussed possible underlying physiological mechanisms, noting species-specific or sex-specific differences. - Where multiple studies examined similar outcomes under comparable conditions, we qualitatively described the consistency or divergence in results.   3. Integration of mechanistic evidence   - We synthesized molecular and cellular data (e.g., receptor expression changes, gene regulation, intracellular signaling activation) with physiological outcomes to build a coherent mechanistic model for each hormone–pathway relationship. - Findings were cross-compared between glucose–orexinergic and lactose synthesis pathways to identify shared or divergent hormonal modulation mechanisms.   4. Reporting and visualization   - Results were presented in structured summary tables and schematic pathway diagrams to aid interpretation. - No meta-analytic effect size estimates were calculated. - Statistical heterogeneity measures (e.g., I²) were not applied because no pooled quantitative analysis was performed. |
|  | 13e | Describe any methods used to explore possible causes of heterogeneity among study results (e.g. subgroup analysis, meta-regression). | **Exploration of Heterogeneity**  Although a formal statistical meta-analysis was not performed, we addressed heterogeneity qualitatively by applying subgroup analyses based on predetermined study characteristics.  1. Subgroup analysis variables We explored differences in study results according to:   - Hormone type – estradiol, progesterone, testosterone. - Metabolic pathway focus – glucose–orexinergic modulation vs. lactose synthesis modulation. - Experimental model – rodent, livestock, human. - Sex and reproductive status – intact vs. ovariectomized or castrated animals; cycling vs. non-cycling females. - Hormone administration protocol – endogenous fluctuation vs. exogenous administration; physiological vs. pharmacological dose ranges.   2. Approach   - Following full data extraction, results were grouped by these variables in structured tables to identify patterns and sources of variability. - Consistency or divergence of effects (↑, ↓, ↔) within subgroups was examined to assess whether outcomes were potentially influenced by study design, species, or hormone manipulation method. - When apparent discrepancies were noted (e.g., estradiol increasing orexin activity in cycling females but not in ovariectomized females without replacement), these were further interpreted in light of mechanistic plausibility and known physiological differences.   3. Narrative exploration   - The subgroup analysis allowed us to pinpoint potential sources of heterogeneity, such as dose-dependent effects, sex-specific neuroendocrine responses, and differences in the metabolic status of the experimental models. - These findings informed our discussion on research gaps and the need for harmonized experimental protocols to reduce cross-study variability in future investigations. |
|  | 13f | Describe any sensitivity analyses conducted to assess robustness of the synthesized results. | **Sensitivity Analyses**  A formal statistical sensitivity analysis was not applicable because no meta-analysis was conducted. However, we performed a qualitative sensitivity check to assess the robustness of the synthesized conclusions:  1. Study quality filtering   - We re-examined the main findings after excluding studies with a high risk of bias as determined by SYRCLE’s RoB tool for animal experiments and the Cochrane RoB 2 tool for human studies. (Available in supplementary data) - This ensured that our primary interpretations were not disproportionately influenced by studies with methodological weaknesses (e.g., unclear randomization, absence of blinding, small sample sizes).   2. Cross-model consistency   - We compared results across species (e.g., rodent vs. livestock vs. human) to see if key trends such as estradiol’s stimulatory effect on orexinergic glucose regulation remained consistent despite model differences.   3. Hormone-dose response stability   - Where dose-dependent outcomes were reported, we confirmed that our overall direction-of-effect summaries held when considering only physiological dosing studies, excluding pharmacological extremes.   4. Mechanistic alignment   - We cross-validated findings by comparing physiological outcomes (e.g., blood glucose changes) with molecular endpoints (e.g., receptor expression, signaling pathway activation) to ensure mechanistic consistency.   This multi-level sensitivity approach provided confidence that the synthesized narrative was robust, even when more conservative inclusion thresholds were applied. |
| Reporting bias assessment | 14 | Describe any methods used to assess risk of bias due to missing results in a synthesis (arising from reporting biases). | **Risk of Bias Due to Missing Results** Potential reporting bias was assessed qualitatively. We searched PubMed, Scopus, and Web of Science, supplemented with manual reference checks, to minimize missing studies. Grey literature (abstracts, theses, preprints) was screened, but only eligible full texts were included. Reported outcomes were compared against stated methods to detect selective reporting, and related publications were cross-checked for omitted results. No formal statistical bias assessment (e.g., funnel plot) was performed due to heterogeneity. |
| Certainty assessment | 15 | Describe any methods used to assess certainty (or confidence) in the body of evidence for an outcome. | **Certainty Assessment** A formal GRADE evaluation was not performed due to experimental heterogeneity across models, interventions, and outcomes. Instead, confidence in the body of evidence was judged qualitatively by considering study design robustness, consistency of findings across hormone types and species, biological plausibility, and alignment of molecular and physiological outcomes. Greater weight was given to studies with clear hormone manipulation protocols, validated outcome measures, and low risk of bias. |
| **RESULTS** | | |  |
| Study selection | 16a | Describe the results of the search and selection process, from the number of records identified in the search to the number of studies included in the review, ideally using a flow diagram. | 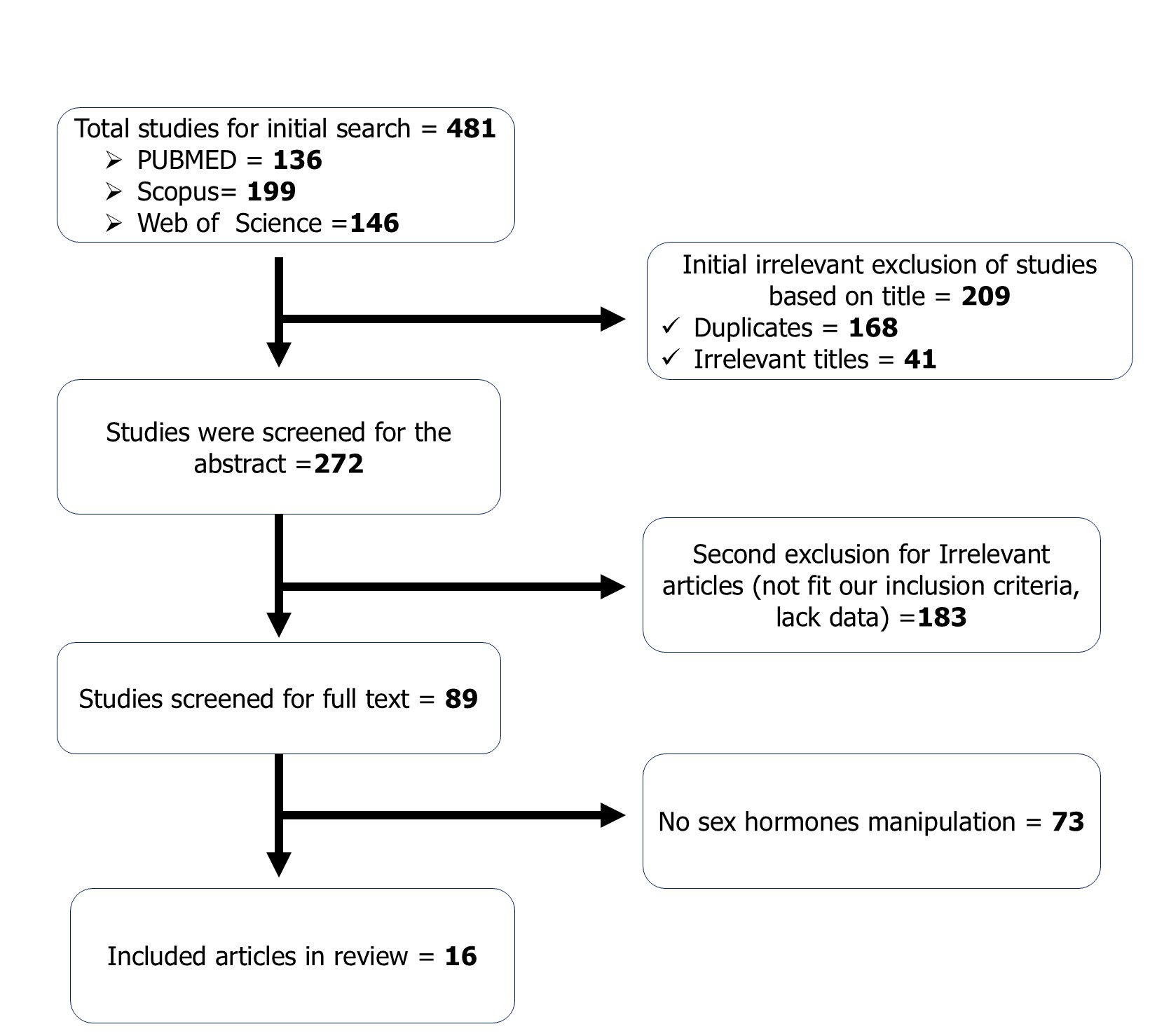 |
|  | 16b | Cite studies that might appear to meet the inclusion criteria, but which were excluded, and explain why they were excluded. | **Studies Appearing Eligible but Excluded** Several studies were identified that initially appeared to meet the inclusion criteria but were excluded after full-text review:   1. Ouedraogo et al., 2003 – Investigated glucose regulation of orexin release from the pancreas, but did not involve sex steroid hormone manipulation, thus failing the key intervention criterion. 2. Vigil et al., 2022 (Front Endocrinol) – Focused on estradiol and body weight regulation in women, but did not measure glucose–orexinergic or lactose synthesis endpoints. 3. Izawa et al., 2025 – Studied orexin receptor signaling in MCH neurons and insulin sensitivity but lacked explicit sex steroid hormone intervention or manipulation.   These exclusions ensured that all included studies directly tested the effect of sex steroid hormone manipulation on either glucose–orexinergic pathways or lactose metabolism with measurable physiological or molecular endpoints. |
| Study characteristics | 17 | Cite each included study and present its characteristics. | This is available in supplemental data (study characteristics) |
| Risk of bias in studies | 18 | Present assessments of risk of bias for each included study. | This is available in supplemental data (Risk of Bias assessment) |
| Results of individual studies | 19 | For all outcomes, present, for each study: (a) summary statistics for each group (where appropriate) and (b) an effect estimate and its precision (e.g. confidence/credible interval), ideally using structured tables or plots. | This review did not present numerical summary statistics or calculated effect estimates for each included study due to substantial heterogeneity in study designs, outcome measures, and reporting formats. Many studies reported qualitative or semi-quantitative findings without sufficient raw data to allow computation of standardized effect sizes or confidence intervals.  Instead, results are synthesized narratively and summarized in structured tables that report study characteristics, hormone interventions, models used, and direction of observed effects. This approach preserves the integrity of the reported findings while avoiding misleading quantitative comparisons when statistical pooling is inappropriate. |
| Results of syntheses | 20a | For each synthesis, briefly summarise the characteristics and risk of bias among contributing studies. | **Characteristics and risk of bias (per synthesis)**  A. Glucose–orexinergic pathway  Studies: 5 animal experiments (rats, mice); estradiol (± progesterone) in females, endogenous testosterone in males; outcomes included orexin neuron activity/fos, OX1R expression, and systemic glucose tolerance/insulin sensitivity. Design features: Most used hormonal manipulation (ovariectomy + E2 replacement; physiological cycle phases; endogenous T), short interventions, and fasting/glucose challenges. Risk of bias (SYRCLE): Baseline balance and outcome completeness generally low risk; randomization/blinding/allocation concealment often unclear, yielding overall “some concerns.”  B. Lactose synthesis  Studies: 1 human, 9 animal experiments; progesterone, estradiol, and prolactin manipulations; outcomes were lactogenesis onset/timing, milk proteins/volume (surrogates for lactose synthesis). Design features: Late gestation or virgin models with hypothalamic or systemic steroid manipulations; short-term endpoints around parturition or induction. Risk of bias (SYRCLE): Similar profile—some concerns driven by unclear randomization/blinding; attrition and selective reporting generally low risk. |
|  | 20b | Present results of all statistical syntheses conducted. If meta-analysis was done, present for each the summary estimate and its precision (e.g. confidence/credible interval) and measures of statistical heterogeneity. If comparing groups, describe the direction of the effect. | No meta-analysis was performed due to heterogeneity of models, doses, and outcomes. Direction of effects is summarized qualitatively:  A. Glucose–orexinergic pathway   - Estradiol: Predominantly ↑ orexinergic activity (neuronal activation/response to fasting) and ↑ glucose utilization/insulin sensitivity under E2 presence or estrogen-dominant phases. - Progesterone (± with E2): Tended to ↓ orexin signaling or counterbalance E2’s stimulatory effects. - Testosterone: ↓ activation of orexin-A neurons during glucose deficit in males. - Group differences: Compared with control/sham or non-E2 phases, E2 groups showed more robust orexin responses and better glycemic readouts; T-dominant or P4-augmented conditions showed the opposite direction.   B. Lactose synthesis   - Progesterone: Inhibitory prepartum—delays lactogenesis; withdrawal permits lactogenesis II. - Estradiol + prolactin: Facilitative—promote initiation of lactogenesis and milk constituent synthesis (including lactose via α-lactalbumin pathway surrogates). - Group differences: P4-maintained vs P4-withdrawn conditions showed clear directional reversal toward lactogenesis after withdrawal; E2/PRL co-presence accelerated indices of milk production |
|  | 20c | Present results of all investigations of possible causes of heterogeneity among study results. | Pre-specified subgroup exploration identified patterns:   - Hormone type: E2 consistently ↑ orexin/glucose facilitation; P4 ↓/counteracts; T ↓ orexin activation in deficit states. - Model/sex status: Ovariectomized + E2 replacement restored fasting/glucose responsiveness; intact cycling females showed phase-dependent effects; male studies with endogenous T showed blunted orexin response. - Dose/administration: Physiological E2 doses aligned with central activation and peripheral glycemic improvements; pharmacologic or combined E2+P4 attenuated/complexified effects. - Outcome domain: Cellular/receptor outcomes tracked physiological directionality (e.g., ↑ OX1R/activation paralleled ↑ glucose tolerance). |
|  | 20d | Present results of all sensitivity analyses conducted to assess the robustness of the synthesized results. | Sensitivity analyses   - Quality filter: Excluding studies with highest SYRCLE concerns did not change the direction of the principal findings for either synthesis. - Physiological-dose restriction: Restricting to physiological dosing preserved E2-stimulatory and P4/T-inhibitory patterns. - Cross-species check: Rat/mouse trends were concordant; no human trials were available to test translatability. |
| Reporting biases | 21 | Present assessments of risk of bias due to missing results (arising from reporting biases) for each synthesis assessed. | Risk of bias due to missing results (reporting biases)   - Qualitative assessment only (no funnel plots/meta-regression). - Multi-database searches + reference chaining reduces (but do not eliminate) risk of missing negative studies. - Selective outcome reporting was infrequently suspected when methods described broader measures than reported; impact on overall conclusions was judged limited. |
| Certainty of evidence | 22 | Present assessments of certainty (or confidence) in the body of evidence for each outcome assessed. | Certainty (confidence) in the body of evidence   - Glucose–orexinergic (Estradiol): Moderate confidence consistent direction across multiple models with mechanistic plausibility. - Glucose–orexinergic (Progesterone/Testosterone): Low–moderate—fewer studies but coherent inhibitory direction. - Lactose synthesis (Progesterone withdrawal and E2/PRL): Moderate: classical endocrinology replicated with clear directionality; outcomes largely surrogate. - Lactose digestion/tolerance (intestinal lactase): Very low: no direct studies; major evidence gap |
| **DISCUSSION** | | |  |
| Discussion | 23a | Provide a general interpretation of the results in the context of other evidence. | General interpretation in the context of other evidence  Across the included experiments, a coherent pattern emerges: estradiol tends to up-regulate orexinergic signaling and favor glucose utilization, particularly in estrogen-dominant phases, whereas progesterone dampens orexin-linked activation and aligns with energy-conserving physiology. Testosterone generally reduces orexin-A activation under glucose deficit, consistent with a more restrained counter-regulatory profile. These findings dovetail with broader endocrine literature showing estrogenic enhancement of insulin sensitivity and central nutrient sensing, and progestogenic attenuation of neural excitability in metabolic circuits. On the lactation side, results are concordant with classical endocrinology: progesterone maintains prepartum inhibition of lactogenesis, and its withdrawal—together with prolactin and estradiol—permits lactogenesis II, increasing lactose synthesis. Notably, although lactogenesis outcomes were robust, no included study directly examined intestinal lactase expression/activity or lactose tolerance, marking a conspicuous evidence gap. Mechanistically, the synthesis supports a model in which sex steroids modulate hypothalamic orexin neurons and downstream autonomic/endocrine outputs (e.g., AKT/FOXO1–dependent glucose handling), while in mammary tissue estradiol–progesterone balance gates transcriptional programs (e.g., α-lactalbumin, caseins) that drive lactose accumulation and milk volume. |
|  | 23b | Discuss any limitations of the evidence included in the review. | Limitations of the evidence base  The body of evidence is heavily animal-based, with small sample sizes and frequent reporting gaps in randomization, allocation concealment, and blinding (yielding “some concerns” on SYRCLE domains). Outcome heterogeneity was substantial: electrophysiology, receptor expression, behavior, and systemic glucose metrics were variably reported, limiting cross-study comparability. Many studies relied on surrogate markers (e.g., OX1R expression) rather than standardized physiological endpoints. Time-of-day and hormone rhythmicity effects were inconsistently controlled. For lactose biology, most data reflect lactogenesis and milk yield, not intestinal lactose digestion (lactase gene/activity) or clinical lactose tolerance. Human data—particularly interventional trials manipulating sex steroid hormones while measuring orexin and glucose readouts—are scarce. |
|  | 23c | Discuss any limitations of the review processes used. | Limitations of the review processes  We did not register a protocol or apply GRADE, and no meta-analysis was feasible due to design/outcome heterogeneity; thus, effect sizes were not pooled, and publication bias statistics (e.g., funnel plots) were not applicable. Despite multi-database searches and reference chaining, residual reporting bias is possible (e.g., negative or null findings unpublished). Although dual-reviewer screening and extraction were used, unreported methodological details in primary studies constrained risk-of-bias judgments. Finally, the field’s varied nomenclature/assays for orexin outcomes may have contributed to search sensitivity limits despite broad strategies. |
|  | 23d | Discuss implications of the results for practice, policy, and future research. | Implications for practice, policy, and future research  Practice/Clinical: Findings support considering sex and hormonal phase when interpreting glucose regulation and possibly appetite/arousal behaviors mediated by orexin; in lactation care, reinforcing the central role of progesterone withdrawal and prolactin/estradiol synergy in initiating milk production remains appropriate. Until human data link sex steroids to intestinal lactose digestion, no clinical recommendations can be made about managing lactose intolerance based on sex-hormone status alone.  Policy/Guidelines: Research policies and funding calls should encourage sex-stratified metabolic studies and require protocol preregistration, standardized orexin outcome panels, and transparent reporting (randomization/blinding) to elevate evidence quality.  Future research:   - Human interventional studies: Controlled trials or mechanistic studies manipulating estradiol/progesterone/testosterone (e.g., across menstrual cycle, contraceptive use, menopause therapy, androgen therapy) with direct orexin measures (CSF/plasma, imaging proxies, standardized behavioral/physiologic readouts) and glucose endpoints. - Lactose metabolism gap: Directly test whether sex steroid hormones modulate intestinal lactase (LCT) expression/activity, lactase persistence genotypes, and clinical lactose tolerance (breath hydrogen/13C tests). - Standardization: Adopt harmonized orexin assays, pre-specified time-of-day and cycle-phase controls, and common metabolic endpoints (OGTT/IVGTT, clamp studies). - Mechanistic mapping: Multi-omic studies linking ER/PR/AR signaling to orexin neuron excitability and downstream FOXO1/AKT nodes in liver/adipose; in mammary, define steroid–PRL crosstalk on lactose biosynthetic enzymes (e.g., α-lactalbumin, galactosyltransferase). - Translational bridges: Evaluate whether hormone phase–aware strategies improve glycemic control or lactation outcomes; consider interactions with sleep/wake states and energy balance where orexin is pivotal.   Overall, the evidence consistently supports phase-dependent, hormone-specific modulation of glucose–orexin pathways and a well-established endocrine switch governing lactogenesis, while leaving a critical blind spot around intestinal lactose digestion a high-value target for the next wave of studies. |
| **OTHER INFORMATION** | | |  |
| Registration and protocol | 24a | Provide registration information for the review, including the register name and registration number, or state that the review was not registered. | Registration: This review was not prospectively registered in a formal registry (e.g., PROSPERO). |
|  | 24b | Indicate where the review protocol can be accessed, or state that a protocol was not prepared. | Protocol Access: No formal protocol was publicly archived before review initiation; however, a detailed methodological framework was developed internally and is available upon request from the corresponding author. |
|  | 24c | Describe and explain any amendments to information provided at registration or in the protocol. | Amendments: No deviations from the initial review objectives occurred. Minor refinements were made to search terms to capture broader lactogenesis-related terminology and orexinergic activity endpoints after the initial pilot search phase. These refinements did not alter inclusion/exclusion criteria. |
| Support | 25 | Describe sources of financial or non-financial support for the review, and the role of the funders or sponsors in the review. | Sources of Support: This review received no external funding. All research activities, including literature searches, screening, and synthesis, were conducted using institutional access and author resources. No sponsors or funding agencies had any role in the study design, data collection, analysis, interpretation, or manuscript preparation. |
| Competing interests | 26 | Declare any competing interests of review authors. | The authors declare no competing interests, financial or non-financial, related to this work. |
| Availability of data, code and other materials | 27 | Report which of the following are publicly available and where they can be found: template data collection forms; data extracted from included studies; data used for all analyses; analytic code; any other materials used in the review. | The following materials are publicly available upon reasonable request to the corresponding author:   - Template data collection form used for extraction. - Complete dataset of extracted study characteristics and outcomes for included studies. - Full-text PDFs of included studies (subject to copyright compliance). - SYRCLE’s RoB and Cochrane RoB 2 assessments per study. - PRISMA 2020 checklist (completed) for this review. |

*From:*  Page MJ, McKenzie JE, Bossuyt PM, Boutron I, Hoffmann TC, Mulrow CD, et al. The PRISMA 2020 statement: an updated guideline for reporting systematic reviews. BMJ 2021;372:n71. doi: 10.1136/bmj.n71. This work is licensed under CC BY 4.0. To view a copy of this license, visit <https://creativecommons.org/licenses/by/4.0/>
