# Supplementary material for: Modulation of lactose synthesis and orexinergic‐glucose pathway by sex steroid hormones
Source: Physiol Rep. 2025 Nov 16;13(22):e70661. doi: 10.14814/phy2.70661 (PMC12620397; doi:10.14814/phy2.70661)
Supplement: Supplementary file 1 — Appendices S1–S4. [file PHY2-13-e70661-s001.zip › Supplementary file S2.docx]

**Search Strategy**

Topic: **Modulation of lactose synthesis and orexinergic-glucose pathway by sex steroid hormones**

**Search Strategy**

**Databases Searched:** PubMed (MEDLINE), Scopus, Web of Science
**Date Last Searched:** July 31, 2025
**Search Period:** From database inception to July 2025
**Languages:** English only
**Search Type:** Boolean keyword + MeSH/EMTREE terms

**1. PubMed (MEDLINE)**

(("sex steroid hormones"[MeSH Terms] OR "estradiol"[MeSH Terms] OR "progesterone"[MeSH Terms] OR "testosterone"[MeSH Terms] OR "gonadal steroids"[MeSH Terms] OR estradiol[tiab] OR progesterone[tiab] OR testosterone[tiab] OR "sex steroid*"[tiab]))

AND

(("orexin"[MeSH Terms] OR "hypocretin"[tiab] OR orexinergic[tiab] OR "OX1R"[tiab] OR "OX2R"[tiab])

OR

("glucose metabolism"[MeSH Terms] OR "gluconeogenesis"[MeSH Terms] OR "glucose uptake"[tiab] OR "insulin sensitivity"[tiab]))

OR

(("lactose"[MeSH Terms] OR "lactase"[MeSH Terms] OR "lactogenesis"[MeSH Terms] OR lactose[tiab] OR lactase[tiab] OR lactogenesis[tiab] OR "milk production"[tiab])

AND

("gene expression"[MeSH Terms] OR "enzyme activity"[MeSH Terms] OR "metabolism"[MeSH Terms]))

**2. Scopus**

(TITLE-ABS-KEY("sex steroid hormone*" OR estradiol OR progesterone OR testosterone OR "gonadal steroid*"))

AND

(TITLE-ABS-KEY(orexin OR hypocretin OR orexinergic OR "OX1R" OR "OX2R" OR "glucose metabolism" OR gluconeogenesis OR "glucose uptake" OR "insulin sensitivity"))

OR

(TITLE-ABS-KEY(lactose OR lactase OR lactogenesis OR "milk production")

AND TITLE-ABS-KEY("gene expression" OR "enzyme activity" OR metabolism))

**3. Web of Science**

TS=("sex steroid hormone*" OR estradiol OR progesterone OR testosterone OR "gonadal steroid*")

AND

TS=(orexin OR hypocretin OR orexinergic OR "OX1R" OR "OX2R" OR "glucose metabolism" OR gluconeogenesis OR "glucose uptake" OR "insulin sensitivity")

OR

TS=(lactose OR lactase OR lactogenesis OR "milk production")

AND TS=("gene expression" OR "enzyme activity" OR metabolism)
