# Supplementary material for: Modulation of lactose synthesis and orexinergic‐glucose pathway by sex steroid hormones
Source: Physiol Rep. 2025 Nov 16;13(22):e70661. doi: 10.14814/phy2.70661 (PMC12620397; doi:10.14814/phy2.70661)
Supplement: Supplementary file 1 — Appendices S1–S4. [file PHY2-13-e70661-s001.zip › Supplementary file S3.docx]

**Risk of bias assessment** for the included studies, applying **SYRCLE’s RoB tool** for animal experiments

**Risk of Bias Assessment – SYRCLE’s RoB Tool (Animal Studies)**

| **Study** | **Sequence Generation** | **Baseline Characteristics** | **Allocation Concealment** | **Random Housing** | **Blinding (Caregivers/ Researchers)** | **Blinding (Outcome Assessment)** | **Incomplete Outcome Data** | **Selective Outcome Reporting** | **Other Bias** | **Overall Risk** |
| --- | --- | --- | --- | --- | --- | --- | --- | --- | --- | --- |
| Kim et al., 2023 | Unclear | Low | Unclear | Low | Unclear | Unclear | Low | Low | Low | **Some Concerns** |
| Takamata et al., 2022 | Low | Low | Unclear | Low | Unclear | Unclear | Low | Low | Low | **Some Concerns** |
| Funabashi et al., 2009 | Low | Low | Unclear | Low | Unclear | Unclear | Low | Low | Low | **Some Concerns** |
| Pu et al., 1998 | Unclear | Low | Unclear | Low | Unclear | Unclear | Low | Low | Low | **Some Concerns** |
| Silveyra et al., 2010 | Low | Low | Unclear | Low | Unclear | Unclear | Low | Low | Low | **Some Concerns** |
| Silveyra et al., 2009 | Low | Low | Unclear | Low | Unclear | Unclear | Low | Low | Low | **Some Concerns** |
| Carón & Deis, 1998 | Unclear | Low | Unclear | Low | Unclear | Unclear | Low | Low | Low | **Some Concerns** |
| López-Fontana et al., 2012 | Low | Low | Unclear | Low | Unclear | Unclear | Low | Low | Low | **Some Concerns** |
| Deis et al., 1989 | Low | Low | Unclear | Low | Unclear | Unclear | Low | Low | Low | **Some Concerns** |
| Bussmann et al., 1983 | Unclear | Low | Unclear | Low | Unclear | Unclear | Low | Low | Low | **Some Concerns** |
| Delbecchi et al., 2005 | Low | Low | Unclear | Low | Unclear | Unclear | Low | Low | Low | **Some Concerns** |
| Bolander & Topper, 1980 | Unclear | Low | Unclear | Low | Unclear | Unclear | Low | Low | Low | **Some Concerns** |
| Murphy et al., 1973 | Unclear | Low | Unclear | Low | Unclear | Unclear | Low | Low | Low | **Some Concerns** |
| Palmiter, 1969 | Unclear | Low | Unclear | Low | Unclear | Unclear | Low | Low | Low | **Some Concerns** |
| McGuire, 1969 | Low | Low | Unclear | Low | Unclear | Unclear | Low | Low | Low | **Some Concerns** |
| Harigaya et al., 1978 | Unclear | Low | Unclear | Low | Unclear | Unclear | Low | Low | Low | **Some Concerns** |

**Summary:**

- **Most studies scored “Low” risk** for baseline characteristics, incomplete data, and selective reporting.
- **Allocation concealment and blinding** were seldom clearly described, leading to a consistent classification of “Some Concerns.”
- There are only 2 human studies, and there were no human RCTs included. So, **Cochrane RoB 2** was not applied.
- The **overall certainty of evidence is moderate**, mainly limited by unclear risk in randomization and blinding domains.
